# Pregnancy as a risk factor for central serous chorioretinopathy: A systematic review and meta‐analysis

**DOI:** 10.1111/aos.70013

**Published:** 2025-09-30

**Authors:** Nathalie Skovgaard Eriksen, Zainab Fakhril‐din, Riccardo Fasana, Rodrigo Anguita, Elon H. C. van Dijk, Lorenzo Ferro Desideri, Jakob Grauslund, Michael Stormly Hansen, Oliver Niels Klefter, Armin Motaabbed, Line Petersen, Prithvi Ramtohul, Mehmet Cem Sabaner, Osman Savran, Miklos Schneider, Yousif Subhi

**Affiliations:** ^1^ Department of Ophthalmology Rigshospitalet Glostrup Denmark; ^2^ Moorfields Eye Hospital NHS Foundation Trust London UK; ^3^ Department of Ophthalmology, Inselspital, University Hospital Bern University of Bern Bern Switzerland; ^4^ Department of Ophthalmology Leiden University Medical Center Leiden The Netherlands; ^5^ Rotterdam Eye Hospital Rotterdam The Netherlands; ^6^ Department for Bio Medical Research University of Bern Bern Switzerland; ^7^ Bern Photographic Reading Center, Inselspital University Hospital Bern Bern Switzerland; ^8^ Department of Ophthalmology Odense University Hospital Odense Denmark; ^9^ Department of Ophthalmology Vestfold Hospital Trust Tønsberg Norway; ^10^ Department of Region Health Research University of Southern Denmark Odense Denmark; ^11^ Department of Clinical Medicine University of Copenhagen Copenhagen Denmark; ^12^ Department of Ophthalmology Hanusch Hospital Vienna Austria; ^13^ Department of Ophthalmology Aarhus University Hospital Aarhus Denmark; ^14^ Department of Clinical Medicine Aarhus University Aarhus Denmark; ^15^ Department of Ophthalmology, Hospital Nord Aix‐Marseille University Marseille France; ^16^ Vitreous Retina Macula Consultants of New York New York New York USA; ^17^ Department of Ophthalmology Bilecik Training and Research Hospital Bilecik Türkiye; ^18^ Department of Ophthalmology Semmelweis University Budapest Hungary

**Keywords:** central serous chorioretinopathy, meta‐analysis, pregnancy, risk factor, systematic review

## Abstract

**Purpose:**

Pregnancy induces significant changes in the body, including increased peripheral and choroidal blood perfusion and an increased systemic corticosteroid level. Here, we systematically reviewed the evidence on pregnancy as a risk factor for developing central serous chorioretinopathy (CSC) and conducted a meta‐analysis to obtain a summary estimate.

**Methods:**

We searched 12 literature databases on 16 February 2025 for epidemiological studies, which evaluated the potential association between pregnancy and CSC. All eligible studies were included for a qualitative review and a meta‐analysis. The meta‐analysis was made using the random‐effects model on the odds ratio (OR) of the association between pregnancy and CSC.

**Results:**

Four studies were eligible for our review. Of the 2766 individuals (1345 patients with CSC, 1391 controls) in these studies, females constituted 26%–64% of study populations in individual studies. The calculated summary estimate of pregnancy as a risk factor for CSC was OR 5.51 (95% CI: 2.12–14.30, *p* = 0.00046). Heterogeneity statistics suggested minimal to no heterogeneity (Cochran's *Q* = 0.72; *I* ^2^ = 0%), the funnel plot was symmetrical and the sensitivity analysis suggested robustness of the estimates.

**Conclusion:**

Pregnancy appears to be a significant risk factor for CSC.

## 1 INTRODUCTION

Central serous chorioretinopathy (CSC) is the fourth most prevalent exudative disease of the macula (Feenstra et al., 2024; Frederiksen et al., 2025). CSC is thought to be a consequence of venous congestion and increased choroidal hydrostatic pressure because of choroidal arteriovenous anastomoses and outflow problems at the vortex veins (Brinks, van Dijk, Meijer, et al., 2022; Cheung et al., 2019; Pauleikhoff et al., 2024; Spaide et al., 2022; Tschuppert et al., 2022). These choroidal changes are also observed as thicker choroidal vessels—pachychoroid—which is a key observation in eyes with CSC (Cheung et al., 2019). The increased hydrostatic pressure induces a serous elevation of the retinal pigment epithelium (RPE) from Bruch's membrane, and the serous fluid then passes through the RPE to form subretinal fluid, which is the key feature of an active CSC.

Male sex is an important demographic risk factor of CSC (Frederiksen et al., 2025; Spaide et al., 1996). However, approximately 20%–30% of cases with CSC occur in females (Frederiksen et al., 2025; Tsai et al., 2013). Another important risk factor for CSC is any exogenous or endogenous cause of increased corticosteroid levels (Haimovici et al., 2004; Holtz et al., 2022; van Dijk et al., 2016; van Haalen et al., 2019). Focusing on a better understanding of epidemiological risk factors may form the basis of a deeper pathophysiological understanding. Pregnancy causes increased blood volume, increased peripheral blood perfusion and increased corticosteroid levels (Brinks, van Dijk, Tsonaka, et al., 2022; Larsen et al., 2025), and pregnancy also leads to an increased subfoveal choroidal thickness (Larsen et al., 2025). Indeed, several reports have reported cases of CSC in pregnant women (Al‐Mujaini & Wali, 2014; Normalina et al., 1998; Olusanya & Oluleye, 2015; Said‐Ahmed et al., 2012; Yu et al., 2021).

In this study, our aim was to systematically review studies that explored the risk of CSC during pregnancy and to conduct a meta‐analysis to understand and summarize the current evidence on this topic.

## 2 METHODS

This systematic review and meta‐analysis was designed following the Cochrane Handbook (Higgins et al., 2024). We reported this study according to the Preferred Reporting Items for Systematic Reviews and Meta‐Analysis (PRISMA) (Moher et al., 2009). Our protocol was registered a priori at PROSPERO (protocol no. CRD420251017093).

### 2.1 Study eligibility criteria and outcomes of interest

Eligible studies were prospective and retrospective observational studies which evaluated an association between active pregnancy and incident CSC as an outcome. Both cross‐sectional and temporal study designs were considered eligible. We only considered studies with original data. Case reports or conference abstracts were not considered eligible for this review. We did not restrict eligibility based on geography or journal, but for practical purposes, only studies in English were considered eligible.

For the definition of the exposure, active pregnancy was defined as an ongoing pregnancy without any restriction on this definition. We did not include studies with only previous pregnancy as the exposure, and in studies reporting both previous and ongoing pregnancies, we only extracted data on the exposure and outcomes based on the ongoing pregnancies.

For the outcome definition, we accepted any definition of CSC that was defined by the individual study authors. However, we required that the clinical diagnosis of CSC was described (i.e. registry‐based studies without any description of how CSC was diagnosed were not deemed eligible). For temporal studies, incident CSC during the active pregnancy was extracted as the outcome. For cross‐sectional studies, prevalent CSC in individuals with active pregnancy was extracted as the outcome.

### 2.2 Information sources and literature search

One trained author (Y.S.) searched the databases *PubMed*, *Embase*, *Cochrane Central*, *BIOSIS Previews*, *Current Contents Connect*, *Data Citation Index*, *Derwent Innovations Index*, *KCI‐Korean Journal Database*, *Preprint Citation Index*, *ProQuest™ Dissertations and Theses Citation Index*, *SciELO Citation Index and Web of Science Core Collection*. All searches were made on 16 February 2025. Literature search documentation of individual databases is provided in File S1.

### 2.3 Study selection, data extraction and risk of bias within studies

One author (Y.S.) screened the title and the abstracts of all records to remove duplicates and obviously irrelevant records. Two authors (N.S.E. and Z.F.‐D.) then independently evaluated the remaining references for eligibility in full text. Reference lists were reviewed to identify additional eligible studies. Disagreements were discussed, and when consensus could not be resolved through discussion, a senior author (Y.S.) provided the final decision. Upon reaching consensus on study selection, data were extracted from individual studies on study design, population characteristics, exposure details and outcomes. Risk of bias within studies was evaluated using relevant items from the Agency for Healthcare Research and Quality (AHRQ) checklist (Zeng et al., 2015). Data extraction and risk of bias evaluation were made independently by two authors (N.S.E. and Z.F.‐D.). Disagreements were discussed, and when consensus could not be resolved through discussion, a senior author (Y.S.) provided the final decision.

### 2.4 Data analysis, data synthesis and risk of bias across studies

Eligible studies were reviewed qualitatively. For meta‐analyses, the risk of CSC from active pregnancy was either extracted as an odds ratio (OR) or calculated as an OR from individual studies using the reported data. OR estimates were calculated from multivariable adjusted analyses when available. Meta‐analyses were conducted using MetaXL 5.3 (EpiGear International, Sunrise Beach, QLD, Australia) for Microsoft Excel 2013 (Microsoft, Redmond, WA, USA) with the random‐effects model to address potential heterogeneity across studies. Heterogeneity was evaluated using Cochran's *Q* and *I* ^2^. A funnel plot was used to evaluate skewness of results and to substantiate potential publication bias. Sensitivity analyses were made by removing each study in turn and by recalculating the summary measure to understand the impact from individual studies on the summary estimates.

## 3 RESULTS

### 3.1 Study selection

**FIGURE 1:**
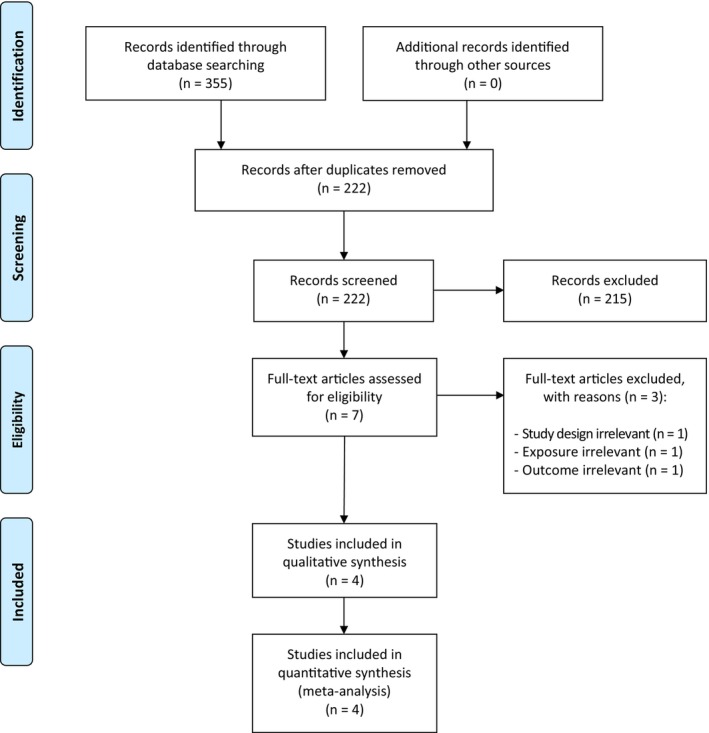
PRISMA flow diagram of study selection process.

Our literature search identified 355 records, of which 133 were duplicates and 215 were obviously irrelevant. The remaining seven records were all retrieved for full‐text examination. Three of these records were not found eligible according to our study eligibility criteria. Four studies were included for the qualitative and quantitative review (Figure 1).

### 3.2 Study characteristics

The four studies summarized data from a total of 2736 individuals (1345 patients with CSC, 1391 controls) (Chatziralli et al., 2017; Ersoz et al., 2019; Haimovici et al., 2004; Karimi et al., 2023). Studies were conducted in Greece (*n* = 1), Iran (*n* = 1), Türkiye (*n* = 1) and the USA (*n* = 1). All four studies were case–control studies. One study was prospective (Chatziralli et al., 2017), and three were retrospective (Ersoz et al., 2019; Haimovici et al., 2004; Karimi et al., 2023). Across studies, the mean age of the study participants ranged between 41 and 49 years. Females constituted the minority of the study population in all four studies. The method for determining pregnancy status varied across studies and was not consistently well‐documented. One study explicitly asked for pregnancy status at the time of clinical evaluation (Chatziralli et al., 2017), and three studies relied on any statement regarding pregnancy retrospectively from medical records (Ersoz et al., 2019; Haimovici et al., 2004; Karimi et al., 2023). For the diagnosis of CSC, all four studies relied on fluorescein angiography and two studies also performed optical coherence tomography. Further details of the study characteristics are outlined in Table 1.

**TABLE 1:** Study characteristics.

References	Country	Study design	Study population	Diagnosis of CSC	Determination of pregnancy
Chatziralli et al. (2017)	Greece	Clinic‐based, prospective, case–control study	183 patients with acute CSC Age 48 ± 4 years 72% males 183 control individuals Age 49 ± 3.3 years 57% males	Acute CSC with a duration of <3 months defined as a localized neurosensory retinal detachment associated with focal leak(s) at the level of RPE based on FA	At the time of ophthalmological examination/diagnosis, all patients and control individuals were asked about active pregnancy
Ersoz et al. (2019)	Türkiye	Clinic‐based, retrospective, case–control study	811 patients with CSC Age 47 ± 10 years 73% males 816 control individuals Age 46 ± 12 years 73% males	‘Classic CSC’ was defined as the presence of serous detachment of the neurosensory retina associated with focal leak(s) at the level of RPE based on FA. Chronic CSC was based on the presence of widespread tracks of RPE atrophy on FAF and focal or diffuse RPE hyperfluorescence on FA with or without serous detachment of the neurosensory retina. OCT was used for evaluation of retinal anatomy. Cases with MNV were excluded	At the time of ophthalmological examination/diagnosis, all patients and control individuals were asked about active pregnancy
Haimovici et al. (2004)	USA	Clinic‐based, retrospective, case–control study	312 patients with CSC Age 45 ± 12 years 74% males 312 control individuals Age 45 ± 13 years 74% males	‘Classic CSC’ was defined as the presence of a localized neurosensory retinal detachment associated with focal leak(s) at the level of RPE based on FA. Chronic CSC was defined as a sensory retinal detachment associated with more widespread areas of RPE atrophy and pigmentary mottling associated with either focal or diffuse RPE hyperfluorescence by FA	Not specified
Karimi et al. (2023)	Iran	Clinic‐based, retrospective, case–control study	39 patients with CSC Age 41 ± 8 years 36% males 80 control individuals Age 41 ± 10 years 55% males	CSC was defined as a localized neurosensory retinal detachment associated with a focal leak at the RPE level, and the diagnosis was based on fundus examination, OCT and FA	At the time of ophthalmological examination/diagnosis, all patients and control individuals were asked about active pregnancy

Abbreviations: CSC, central serous chorioretinopathy; FA, fluorescein angiography; FAF, fundus autofluorescence; MNV, macular neovascularization; OCT, optical coherence tomography; RPE, retinal pigment epithelium.

The association between pregnancy and CSC was evaluated using an unadjusted analysis in one study (Karimi et al., 2023) as there were no cases of pregnancy in either the study or the control group. An adjusted analysis was made in the three other studies. Adjustments were made using multivariable analysis. Details of adjustments made in the statistical analyses of the potential association between pregnancy and CSC are summarized in Table 2.

**TABLE 2:** Adjustments made in the statistical analyses of the potential association between pregnancy and central serous chorioretinopathy.

References	Adjustments made in statistical analyses
Chatziralli et al. (2017)	Multivariable analysis was made as a stepwise multivariable model with backward‐selection approach which in the final model only included the statistically significant variables. The analysis for pregnancy was performed in the female subset of the participants
Ersoz et al. (2019)	Multivariable analysis with backward conditional logistic regression analyses on a subsample of female study participants
Haimovici et al. (2004)	Stepwise logistic regression with case status as the outcome was performed. Predictors with a *p* value of 0.15 or less in univariate analyses, as well as predictors previously noted in the literature, were included as candidate variables in the multivariable stepwise analyses. *p* Value entry and exit criteria of 0.15 were used for the stepwise model
Karimi et al. (2023)	No adjustments were made (zero pregnant cases in both study and control group)

### 3.3 Results of individual studies

Chatziralli et al. (2017) studied 183 patients with acute CSC and 183 healthy controls and found that pregnancy was independently associated with a significantly increased risk of CSC, even after multivariable adjustment. Ersoz et al. (2019) studied 811 patients with any CSC and 816 healthy controls and found that pregnancy was independently associated with a significantly increased risk of CSC. Haimovici et al. (2004) studied 312 cases with any CSC and 312 controls with ophthalmic conditions other than CSC and reported a statistically significant association between pregnancy and CSC, with 18 cases versus only two controls reporting pregnancy at the time of evaluation. Karimi et al. (2023) had a smaller study population of 39 patients with CSC and 80 controls, of which none were pregnant. The risk evaluation of pregnancy was therefore based on zero cases in each group (Karimi et al., 2023).

### 3.4 Risk of bias within studies

Our risk of bias evaluation of individual studies showed that all four studies clearly defined the data source and the applied eligibility criteria. The time period of the study was clearly defined in three studies. Consecutive recruitment was performed in two studies. No studies described any quality assurance processes to confirm the presence of pregnancy (i.e. additional data regarding the pregnancy, ultrasonography or blood sampling). Our risk of bias evaluation of individual studies is presented in detail in Table 3.

**TABLE 3:** Risk of bias within individual studies.

References	Defines source	Eligibility criteria	Time period	Consecutive recruitment	Quality assurance
Chatziralli et al. (2017)	Yes	Yes	Yes	Yes	No
Ersoz et al. (2019)	Yes	Yes	Yes	Unclear	No
Haimovici et al. (2004)	Yes	Yes	Unclear	No	No
Karimi et al. (2023)	Yes	Yes	Yes	Yes	No

*Note*: Studies are assessed on relevant items from the Agency for Healthcare Research and Quality checklist. Defines source: Defines the source of information. Eligibility criteria: Lists inclusion and exclusion criteria or refers to previous publications. Time period: Indicates time period used for identifying participants. Consecutive recruitment: Indicates whether or not subjects were consecutively recruited for the study. Quality assurance: Describes any assessments undertaken for quality assurance purposes.

### 3.5 Meta‐analysis on pregnancy as a risk factor for central serous chorioretinopathy

All four studies of 1345 patients with CSC and 1391 controls were eligible for the meta‐analysis. The calculated summary estimate of pregnancy as a risk factor for CSC was OR 5.51 (95% CI: 2.12–14.30, *p* = 0.00046) (Table 4). Although heterogeneity statistics suggested minimal to no heterogeneity (Cochran's *Q* = 0.72; *I* ^2^ = 0%) and the funnel plot was symmetrical (File S2), these findings should be interpreted with caution due to the limited number of included studies. With only four studies, the power to detect true heterogeneity is low. Moreover, clinical and methodological differences across studies—such as varying definitions of pregnancy, diagnostic criteria for CSC and retrospective data collection—may still introduce underlying heterogeneity not captured by statistical measures. The sensitivity analysis found only minor changes to the summary estimate when omitting studies in turn (OR range: 4.93–6.31) without loss of statistical significance, which indicated the robustness of the meta‐analytical results (File S3).

**TABLE 4:** Meta‐analysis on pregnancy as a risk factor for central serous chorioretinopathy.

References	OR	95% CI	Study weight (%)
Chatziralli et al. (2017)	4.81	1.13–16.80	50
Ersoz et al. (2019)	8.47	1.02–69.07	20
Haimovici et al. (2004)	7.10	1.00–50.70	24
Karimi et al. (2023)	1.43	0.03–74.53	6
Summary estimate	**5.51**	**2.12–14.30**	**100**

*Note*: Bold values indicate summary estimates.

Abbreviations: CI, confidence interval; OR, odds ratio.

## 4 DISCUSSION

The well‐established risk factors for CSC are male sex (OR: 1.9–2.2), corticosteroid use (OR: 2.2–7.0) and psychological stress (OR: 2.7–13.3) (Chatziralli et al., 2017; Karimi et al., 2023). Since CSC predominantly affects males between 20 and 50 years of age, pregnancy as a risk factor in individuals of female sex is a lesser issue from an epidemiological point of view. However, for individual patients, this is important. Our systematic review and meta‐analysis demonstrate that pregnancy is a significant risk factor for developing CSC with an odds ratio (OR) of 5.51.

Increasing evidence suggests that CSC in women, although less frequent, presents distinct characteristics. Studies have shown that women with CSC may have a higher risk of developing macular neovascularization, a severe complication that can worsen visual prognosis and for which an intense treatment schedule is usually required (Bousquet et al., 2022; Sahoo et al., 2024). Moreover, women who develop CSC generally present at an older age and often experience a more chronic disease course (Liew et al., 2013; Sahoo et al., 2024; Yoneyama et al., 2022). In the studies included in this analysis, the mean age of the study participants ranged between 41 and 49 years, which includes both sexes. This age range has to be considered to be at the upper end of the reproductive spectrum and therefore might not be representative for pregnant women in general. Still, an intriguing speculation could be that this could suggest that pregnancy at an older age could increase the risk of CSC. One retrospective cohort study of the clinical characteristics of pregnancy‐associated CSC in a Chinese population showed that these pregnant patients were 35 ± 4 years old (Yu et al., 2021).

Pregnancy is a temporary physiological state characterized by complex hormonal and vascular changes. Endogenous corticosteroids rise significantly during pregnancy, as much as four‐fold, as part of the hypothalamic–pituitary–adrenal axis activation (Bousquet et al., 2022; Mastorakos & Ilias, 2003; Sandman et al., 2006; Schock et al., 2016). Fluctuation in sex hormones during pregnancy represents another potential mechanism implicated in the pathogenesis of CSC (Brinks, van Dijk, Tsonaka, et al., 2022). Brinks, van Dijk, Tsonaka, et al. (2022) found no significant difference in total testosterone or oestradiol levels in males and females with active CSC compared to controls. However, they found that women with CSC had reduced levels of sex hormone‐binding globulin (SHBG) and a higher free testosterone‐to‐oestradiol ratio. Since SHBG limits the bioavailability of androgens, reduced SHBG during pregnancy may enhance androgen‐mediated vascular effects in the choroid. Oral contraceptives, which increase SHBG, have been proposed to reduce CSC risk, although data remain sparse (Brinks, van Dijk, Tsonaka, et al., 2022). This hormonal imbalance may be particularly relevant in postmenopausal women, who naturally experience declines in SHBG. This may help explain why women with CSC tend to be older at the time of diagnosis (Liew et al., 2013; Sahoo et al., 2024; Yoneyama et al., 2022). In addition to these hormonal changes, pregnancy also induces increased blood volume and cardiac output, augmenting choroidal blood flow and hydrostatic pressure, which may promote choroidal congestion and hyperpermeability (Feenstra et al., 2024). Subfoveal choroidal thickness has also been shown to increase during pregnancy and normalize again postpartum (Larsen et al., 2025). In line with these findings, progesterone, the dominant hormone of pregnancy, peaks in the 3rd trimester (Sundström‐Poromaa et al., 2020), exerts potent vasodilatory effects and increases systemic fluid retention (Sundström‐Poromaa et al., 2020), both of which may contribute to choroidal hyperpermeability and the pathogenesis of CSC during gestation.

In the majority of reported cases of pregnancy‐associated CSC, the subretinal fluid resolves spontaneously postpartum (Al‐Mujaini & Wali, 2014; Normalina et al., 1998; Olusanya & Oluleye, 2015; Said‐Ahmed et al., 2012; Yu et al., 2021). In these cases, the occurrence of CSC peaked during the third trimester, which coincides with peak levels of circulating hormones such as corticosteroids and oestrogens. Following delivery, the levels of these hormones decline, supposedly contributing to the resolution of subretinal fluid in most patients (Quillen et al., 1996; Yu et al., 2021). However, a small number of cases may progress to more chronic forms, potentially resulting in permanent reduction in vision, as described in a case report by Maggio et al. (2015). In this case, spontaneous regression of the subretinal fluid was observed after delivery, but no improvement in visual acuity was seen because of severe atrophic macular changes (Maggio et al., 2015).

Further research about pregnancy complications and the risk of CSC is warranted. However, pregnant women in general pose diagnostic and therapeutic challenges for both scientific and ethical reasons, and therefore, the evidence remains low. Regarding the diagnosis and treatment of CSC, standard imaging modalities like fluorescein angiography and treatments including photodynamic therapy or intravitreal anti‐vascular endothelial growth factor therapy are generally contraindicated due to potential fetal risks (Kornblau & El‐Annan, 2019; Polizzi & Mahajan, 2015). It should be noted that female gender is previously shown to predict less effective response from PDT treatment for CSC (Gawęcki et al., 2025). However, non‐invasive imaging such as OCT can aid the diagnosis, and case reports describe successful use of focal laser photocoagulation and subthreshold micropulse laser during pregnancy (Narayanan et al., 2015; Ochinciuc et al., 2022). These treatment modalities should be considered, especially when early treatment may be preferred (Feenstra et al., 2024).

Limitations should be noted when interpreting the findings of this systematic review and meta‐analysis. The small number of studies and the limited geographic diversity challenge the certainty of the findings. Reliance on retrospective data in three of the four studies increases the risk of recall and detection bias. Inconsistent documentation of pregnancy status and differences in the classification of CSC subtypes further limit the precision of the exposure‐outcome assessment. Finally, the absence of data in reviewed studies regarding details of the pregnancy and presence of any pregnancy‐related complications limited our ability to explore a potential confounder effect. For example, hypertensive chorioretinopathy, which mimics CSC, has been reported in cases with pregnancy‐related hypertension or HELLP syndrome (AlTalbishi et al., 2015; Teodoru et al., 2023). This aspect remains to be studied in more detail in future studies.

In conclusion, while CSC remains predominantly a disease seen in males, our analysis of the four available studies shows that pregnancy is a significant risk factor for CSC in women. The condition, though rare in women, warrants clinical awareness, especially in patients presenting with visual symptoms in the third trimester. Further research is needed to elucidate the contribution of gestational age and pregnancy complications to CSC pathogenesis and to develop evidence‐based management strategies tailored for this important patient population.

## CONFLICT OF INTEREST STATEMENT

Author J.G. declares to have received speaker's fees from Allergan, Bayer, Novartis and Roche, and to have served as an advisory board member for Allergan, Apellis, Bayer, Novartis and Roche, not related to this work. Author M.S.H. declares to have received speaker's fees from Roche. Author M.S. declares to have served as an advisory board member for Roche and Bayer, and acted as a consultant for AbbVie, received speaker fees from AbbVie and Roche and received travel grants from AbbVie, Bayer and Roche, not related to this work. Author Y.S. declares to have received speaker's fees from Bayer and Roche, to have served as an advisory board member for Astellas and to be the inventor of a patent related to biomarkers for polypoidal choroidal vasculopathy (WO2020007612A1), not related to this work. All other authors declare that no potential conflicts of interest exist in relation to this work.

## Supporting information


File S1.

File S2.

File S3.
