# Supplementary material for: Pregnancy as a risk factor for central serous chorioretinopathy: A systematic review and meta‐analysis
Source: Acta Ophthalmol. 2025 Sep 30;104(3):259–66. doi: 10.1111/aos.70013 (PMC13058684; doi:10.1111/aos.70013)

**Supplementary file 1.** Details of the literature search in individual databases.

**PubMed:**


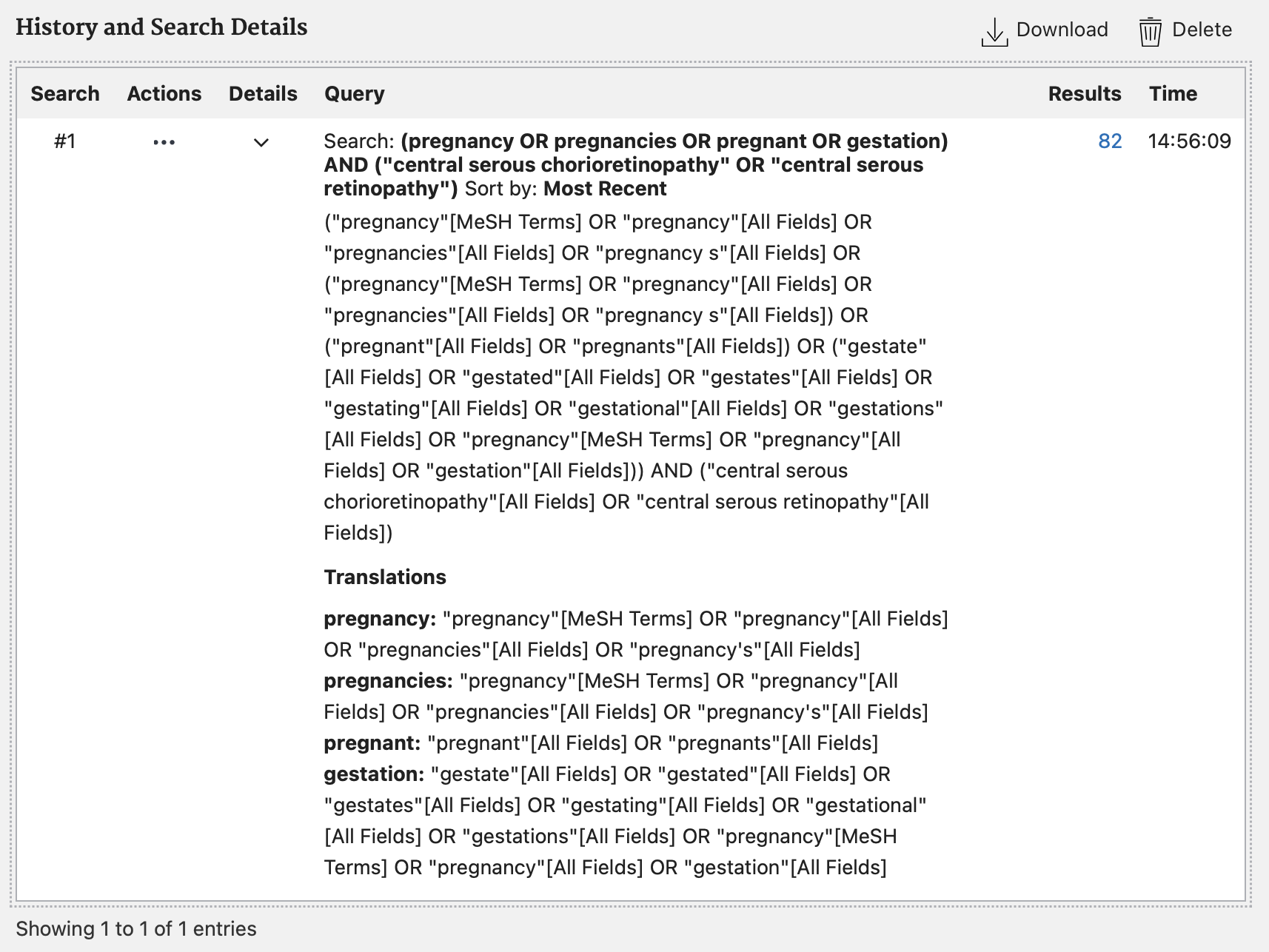


**Embase:**

Embase <1974 to 2025 February 14>

1 pregnancy.mp. [mp=title, abstract, heading word, drug trade name, original title, device manufacturer, drug manufacturer, device trade name, keyword heading word, floating subheading word, candidate term word] 1062961

2 pregnancy/ 721596

3 pregnancies.mp. [mp=title, abstract, heading word, drug trade name, original title, device manufacturer, drug manufacturer, device trade name, keyword heading word, floating subheading word, candidate term word] 156244

4 pregnant.mp. [mp=title, abstract, heading word, drug trade name, original title, device manufacturer, drug manufacturer, device trade name, keyword heading word, floating subheading word, candidate term word] 343655

5 gestation.mp. [mp=title, abstract, heading word, drug trade name, original title, device manufacturer, drug manufacturer, device trade name, keyword heading word, floating subheading word, candidate term word] 205610

6 "central serous chorioretinopathy".mp. [mp=title, abstract, heading word, drug trade name, original title, device manufacturer, drug manufacturer, device trade name, keyword heading word, floating subheading word, candidate term word] 3850

7 central serous retinopathy/ 4693

8 "central serous retinopathy".mp. [mp=title, abstract, heading word, drug trade name, original title, device manufacturer, drug manufacturer, device trade name, keyword heading word, floating subheading word, candidate term word] 4815

9 1 or 2 or 3 or 4 or 5 1206176

10 6 or 7 or 8 5285

11 9 and 10 133

**Cochrane Central:**

Search Name:

Date Run: 17/02/2025 21:05:31

Comment:

ID Search Hits

#1 pregnancy OR pregnancies OR pregnant OR gestation 100249

#2 "central serous chorioretinopathy" OR "central serous retinopathy" 337

#3 #1 AND #2 9

**Web of Science Core Collection, BIOSIS Previews, Current Contents Connect, Data Citation Index, Derwent Innovations Index, KCI-Korean Journal Database, Preprint Citation Index, ProQuest^TM^ Dissertations & Theses Citation Index, and SciELO Citation Index:**


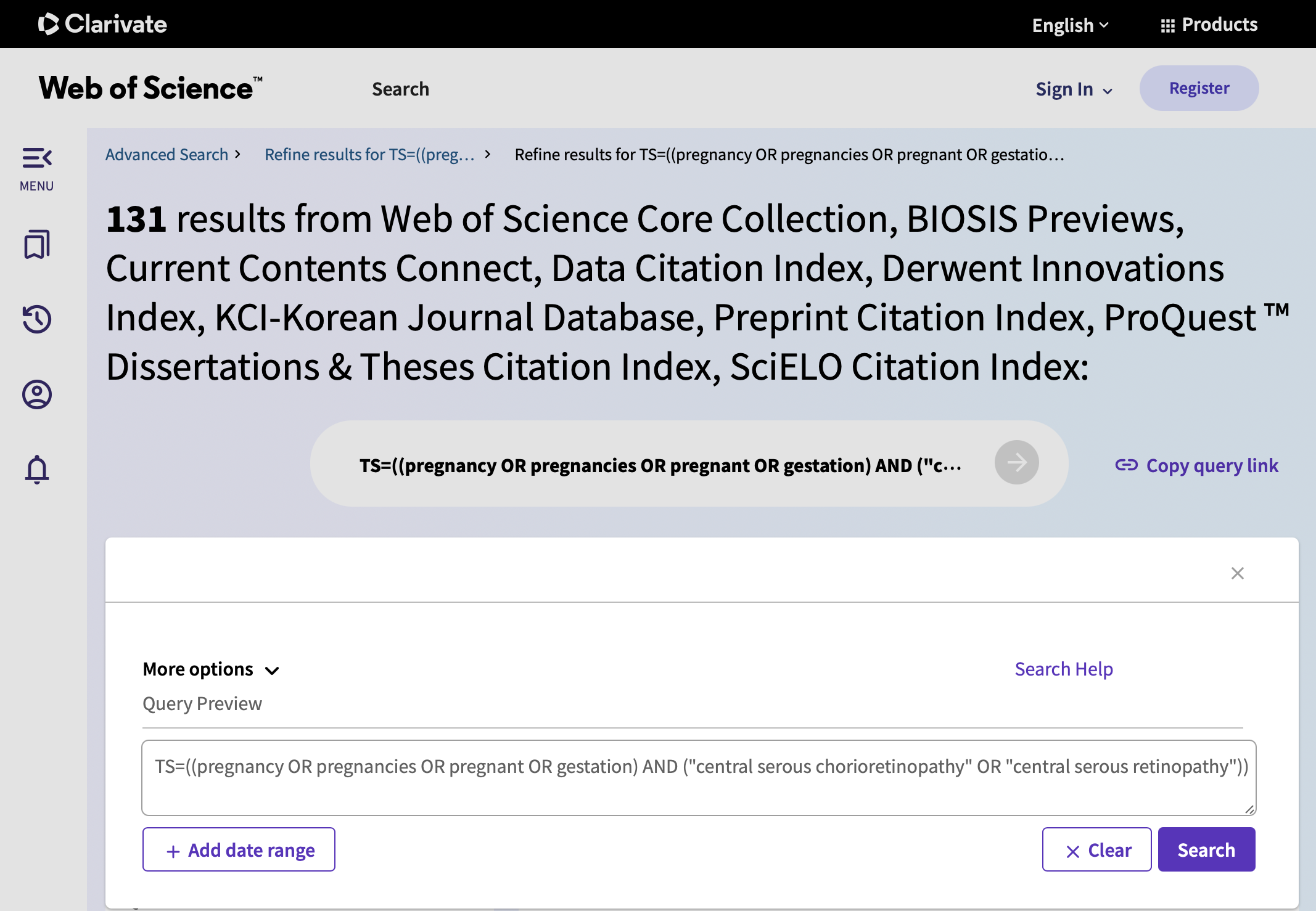

Supplement: Supplementary file 1 — File S1. [file AOS-104-259-s001.docx]
