# Supplementary material for: Pregnancy as a risk factor for central serous chorioretinopathy: A systematic review and meta‐analysis
Source: Acta Ophthalmol. 2025 Sep 30;104(3):259–66. doi: 10.1111/aos.70013 (PMC13058684; doi:10.1111/aos.70013)

**Supplementary file 2.** Funnel plot for risk of bias evaluation across studies for the meta-analysis of pregnancy as a risk factor for central serous chorioretinopathy.


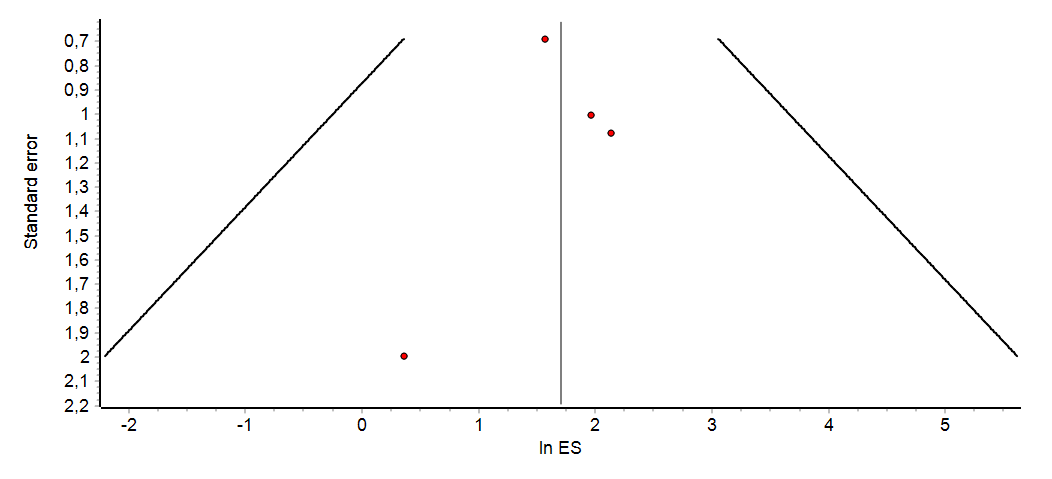

Supplement: Supplementary file 2 — File S2. [file AOS-104-259-s002.docx]
