# Supplementary material for: Pregnancy as a risk factor for central serous chorioretinopathy: A systematic review and meta‐analysis
Source: Acta Ophthalmol. 2025 Sep 30;104(3):259–66. doi: 10.1111/aos.70013 (PMC13058684; doi:10.1111/aos.70013)
Supplement: Supplementary file 3 — File S3. [file AOS-104-259-s003.docx]

**Supplementary file 3.** Sensitivity analysis of the meta-analysis of pregnancy as a risk factor for central serous chorioretinopathy.

| Excluded study | Pooled ES | LCI 95% | HCI 95% | Cochran’s Q | I^2^ |
| --- | --- | --- | --- | --- | --- |
| Chatziralli et al. (2017) | 6.31 | 1.64 | 24.29 | 0.64 | 0.00 |
| Ersoz et al. (2019) | 4.93 | 1.69 | 14.37 | 0.52 | 0.00 |
| Haimovici et al. (2004) | 5.09 | 1.71 | 15.17 | 0.64 | 0.00 |
| Karimi et al. (2023) | 6.00 | 2.24 | 16.04 | 0.23 | 0.00 |
